# Unique Biological Properties of Catalytic Domain Directed Human Anti-CAIX Antibodies Discovered through Phage-Display Technology

**DOI:** 10.1371/journal.pone.0009625

**Published:** 2010-03-10

**Authors:** Chen Xu, Agnes Lo, Anuradha Yammanuru, Aimee St. Clair Tallarico, Kristen Brady, Akikazu Murakami, Natasha Barteneva, Quan Zhu, Wayne A. Marasco

**Affiliations:** 1 Department of Cancer Immunology and AIDS, Dana-Farber Cancer Institute, Harvard Medical School, Boston, Massachusetts, United States of America; 2 Immune Disease Institute and Program in Cellular and Molecular Medicine, Children's Hospital, Harvard Medical School, Boston, Massachusetts, United States of America; 3 Department of Medicine, Harvard Medical School, Boston, Massachusetts, United States of America; 4 Department of Pathology, Harvard Medical School, Boston, Massachusetts, United States of America; Griffith University, Australia

## Abstract

Carbonic anhydrase IX (CAIX, gene G250/MN-encoded transmembrane protein) is highly expressed in various human epithelial tumors such as renal clear cell carcinoma (RCC), but absent from the corresponding normal tissues. Besides the CA signal transduction activity, CAIX may serve as a biomarker in early stages of oncogenesis and also as a reliable marker of hypoxia, which is associated with tumor resistance to chemotherapy and radiotherapy. Although results from preclinical and clinical studies have shown CAIX as a promising target for detection and therapy for RCC, only a limited number of murine monoclonal antibodies (mAbs) and one humanized mAb are available for clinical testing and development. In this study, paramagnetic proteoliposomes of CAIX (CAIX-PMPLs) were constructed and used for anti-CAIX antibody selection from our 27 billion human single-chain antibody (scFv) phage display libraries. A panel of thirteen human scFvs that specifically recognize CAIX expressed on cell surface was identified, epitope mapped primarily to the CA domain, and affinity-binding constants (KD) determined. These human anti-CAIX mAbs are diverse in their functions including induction of surface CAIX internalization into endosomes and inhibition of the carbonic anhydrase activity, the latter being a unique feature that has not been previously reported for anti-CAIX antibodies. These human anti-CAIX antibodies are important reagents for development of new immunotherapies and diagnostic tools for RCC treatment as well as extending our knowledge on the basic structure-function relationships of the CAIX molecule.

## Introduction

Renal cell carcinoma (RCC) accounts for ∼3% of all adult malignancies. Each year in the Unites States there are >30,000 newly diagnosed cases and more than 10,000 deaths [1]–[3]. RCC is resistant to virtually all conventional modes of treatment, such as radiotherapy and chemotherapy [2], [4]–[6], reinforcing the urgent need for developing new therapeutic strategies. RCC is a clinicopathologically heterogeneous disease, traditionally subdivided into clear cell, granular cell, papillary, chromophobe, spindle cell, cystic, and collecting duct carcinoma subtypes based on morphological features according to the WHO International Histological Classification of Kidney Tumors [7] with clear cell RCC as the most common adult renal neoplasm, representing 70% of all renal neoplasm. RCC is one of the few tumors where spontaneous regression of metastatic disease has been documented after tumor nephrectomy, treatment with placebo in phase III trials or after inflammatory or infectious events [8], [9]. These observations have provided strong evidence of the importance of the immune system in the control of this cancer. Therefore, much attention has been focused on immunotherapeutic modalities for the treatment of RCC.

Important prerequisites for a successful immunotherapy include (a) identification of tumor-associated surface antigens that are not expressed in normal tissues, and (b) discovery of highly specific antibodies to such antigens. The carbonic anhydrase IX (CAIX) is one of the most studied surface antigens in RCC. The gene for CAIX is located on chromosomes 9p12 to 13 and encodes a transmembrane protein that binds zinc and has CA activity [10]–[12]. It is N-glycosylated, and forms oligomers in the nonreduced state [13], [14]. Sequence analysis of the predicted CAIX protein shows that it contains a signal peptide (aa 1–37), an extracellular domain (ECD, aa 38–414) that consists of a carbonic anhydrase region located close to the plasma membrane (CA, aa 135–391) and a region between the signal peptide and the CA domain (PG, aa 53–111) named after its significant homology (38% identity) with a keratin sulfate attachment domain of a human large aggregating proteoglycan, aggrecan [15], a hydrophobic transmembrane region of 20 amino acids (aa 415–434) and a small C-terminus cytoplasmic portion of 25 amino acids (aa 435–459). While CAIX appears at malignant transformation and stains positive in about 95% of clear cell RCC specimens as well as in most renal cell metastases, no CAIX was detected on the surface of normal adult or fetal tissues except its cytoplasmic expression in the epithelium of the bile ducts and small intestine together with the mucous cells of the gastric epithelium [16]. This restricted surface expression of CAIX makes it an excellent target for RCC immunotherapy.

The functional significance of CAIX expression in tumorigenesis remains unclear. CAIX expression can induce malignant phenotype in murine NIH 3T3 fibroblasts, suggesting that CAIX may play a role in control of cell proliferation and transformation [10]. Possible relevance of CAIX to the regulation of cell interactions and proliferation in RCC oncogenesis has been suggested by its cell density-regulated expression and association with tumors [17], [18]. CAIX expression has been shown to modulate E-cadherin-mediated cell adhesion via interaction with β-catenin [19]. Such destabilization of E-cadherin-caterin interactions results in reduced cell adhesion that is associated with tumorigenesis and invasion [20], [21]. In Von Hippel-Landau (VHL) gene-defective tumors, CAIX is overexpressed [22] and may contribute to the tumor microenvironment by maintaining extracellular acidic pH and helping cancer cells grow and metastasize [23], [24].

To date, two mAbs against human CAIX have been extensively studied. In 1986, Oosterwijk and colleagues reported the isolation of a murine IgG1 mAb termed G250, which preferentially recognized a surface antigen that was associated with 46 of 47 primary RCC and 7 of 8 RCC metastases while no staining observed in normal kidney tissues [16]. In 1992, Pastoreková and colleagues reported on a murine IgG2b mAb termed M75 with similar properties (13). The antigen recognized by G250 and M75 was cloned, found to be identical, and named CAIX/MN/G250 [10], [12]. The M75 epitope has been localized to the PG domain of CAIX [25]. In order to avoid the human anti-mouse antibody (HAMA) responses seen with antibodies of murine origin, a chimeric form of G250 (mouse Fv and human IgG1 Fc) termed cG250 or WX-G250 was developed [26] and tested for its safety and tolerance in several clinical trials. The results showed that the cG250 was well tolerated and safe with certain clinical benefits such as disease stabilization [27]–[30] and increased median survival rates (www.wilex.com). As the level of CAIX expression has been shown to correlate with patient's prognosis, survival, and response to IL-2 therapy [31], [32], anti-CAIX mAbs G250 and M75 also have great promise as diagnostic/prognostic tools that could aid with treatment decisions [31], [33]–[36].

Although with significant diagnostic and therapeutic potential for RCC, only a limited number of mouse anti-human CAIX antibodies have been identified [13], [16], [37] and essentially only one humanized anti-CAIX mAb has been evaluated in the clinical setting. As such, an interesting but to our knowledge unanswered question is whether antibodies to other epitopes of the CAIX would be more effective in recruiting effecter cells or able to antagonize any effect on cell proliferation and transformation that may be mediated through CAIX. In an effort to develop effective therapeutic reagents for RCC, a panel of high affinity human monoclonal antibodies against CAIX was selected from our 27 billion-member human scFv-phage display library. These antibodies were extensively evaluated by measurement of affinity binding constants, epitope mapping, inhibition of the CA enzymatic activity and capacity to induce CAIX internalization.

## Methods

### Cell Culture

Human renal clear cell carcinoma (RCC) cell line sk-rc-52 (CAIX positive) and sk-rc-59 (CAIX negative) were obtained from Dr. Gerd Ritter (Memorial Sloan-Kettering Cancer Center, New York, NY). These lines, as well as human embryonic kidney cell line 293T (ATCC) were grown in Dulbecco's Modified Eagle's Medium (Life Technologies) supplemented with 10% (v/v) heat-inactivated fetal calf serum (FCS), 100 IU/ml penicillin and 100 µg/ml streptomycin (complete DMEM) at 37°C with 5% CO2.

### Molecular Cloning and Expression of CAIX Proteins and Anti-CAIX Antibodies

Full length or different domains of CAIX cDNA were cloned into mammalian or bacterial expression vector and expressed alone, as human IgG-Fc or bacterial GST fusion proteins. Likewise, anti-CAIX scFv antibodies were also subcloned and expressed as soluble scFv format in bacteria or as scFv-Fc fusion in mammalian cells. Details for subcloning and expression of the recombinant proteins as well as establishing CAIX expressing stable cell line are provided in Text S1.

### Selection of Anti-CAIX Single-Chain Antibodies from Nonimmune Human scFv Phage Display Library with PMPL Panning

The antibody selection procedure including CAIX-PMPL preparation and characterization, panning, cell-based ELISA screening, and confirmation by flow cytometric analysis as well as DNA sequencing were basically performed following the procedure described previously [38], [39]. Additional details are provided in Text S1.

### Kinetic Analysis of Binding Interactions between Anti-CAIX scFvFcs and CAIX-ECD-C9 by Surface Plasmon Resonance (SPR)

The kinetic interactions between anti-CAIX scFvFcs and CAIX-ECD-C9 were measured by SPR using Biacore T100. Sensor chip CM4, amine coupling reagents [N-ethyl-N'-dimethylaminopropyl carbodimide, EDC; N-hydroxysuccinimide, NHS; and 1 M ethanolamine HCl (pH 8.5)], human antibody capture kit and HBS-EP+ buffer were obtained from GE Healthcare. Specific procedure is described in the Text S1. Data analysis was performed using Biacore T100 evaluation software (v1.1.1; GE Healthcare). Data were double referenced by subtraction of reference surface data and blank buffer sample data from all the data sets. The affinity and rate constants of the antigen-antibody interactions were determined by global analysis using a simple 1∶1 langmuir binding model (A+B ⇔ AB), provided by the T100 evaluation software. However, the data sets of G6, G125 and G9 were analyzed using a 2-state model (A+B ⇔ AB ⇔ AB*) since they indicate a more complex mechanism of interaction. Rmax was set as a local parameter to account for the changing ligand density due to the capture of fresh ligand in each binding cycle.

### Epitope Mapping of Anti-CAIX scFvFc Antibodies

#### Biotinylation of CAIX-Fc fusion proteins and anti-CAIX scFvFc antibodies

All fusion proteins and antibodies were labeled with Pierce EZ-Link Sulfo-NHS-Biotinylation kit (Cat. No. 21425) according to the manufacturer's instructions. The biotinylated proteins were examined by SDS-PAGE and the concentration determined by Micro BCA protein assay kit (Pierce).

#### ELISA with purified biotinylated CAIX-Fc fusion proteins

5 ug/ml of 14 different anti-CAIX scFvFc in PBS were coated onto a 96 well maxi-sorp plate at 4°C overnight. An irrelevant scFvFc antibody 1567 or PBS only was used as negative controls. After blocking with 2%BSA/4%milk/PBS for one hour at room temperature (RT) and washing with ELISA washing buffer (0.05% Tween 20 in PBS, PBST) four times, equal molar concentrations of biotinylated CAIX-ECD-Fc (6 µg/ml), CAIX-CA-Fc (5 ug/ml) or CAIX-PG-Fc (3 µg/ml) in 1%BSA/PBS was added and incubated for 1 hour at RT. Specific binding of antigens was detected by incubation with HRP-labeled streptavidin followed by addition of TMB substrate for color development. OD_450_ values were determined by Bio-Rad microplate spectrophotometer.

#### ELISA with CA-GST and PG-GST fusion proteins

96-well plate was coated with 50 µl PBS containing 5 µg/ml CA-GST or PG-GST fusion proteins at 4°C overnight. HA-GST, a fusion protein between HA1 domain of hemagglutinin (HA) of A/Thailand/2(SP-33)/2004 (H5N1) (Geneback Accession # AAS65618) and GST, was cloned, purified and used as a negative control. Upon blocking with 200 µl 4% non-fat milk, 50 µl of 5 µg/ml of anti-CAIX scFvFc antibodies were added to each well and incubated for 1 hr at RT. Irrelevant anti-CXCR4 scFvFc antibody X33 or same volume of PBS was used as negative controls. Specific binding was detected with 50 µl HRP-labeled goat-anti-human Fc IgG and color development was the same as described above.

#### Competition binding to CAIX-ECD-Fc protein

96-well plate was coated with 50 µl PBS containing 5 µg/ml CAIX-ECD-Fc fusions proteins at 4°C overnight. After blocking with 200 µl 4% non-fat milk, 50 µl of 5 µg/ml of biotinylated anti-CAIX scFvFcs were added to each well in the presence or absence of 250 ng of a competing unlabeled antibody, and incubated for 1 hr at RT. Human anti-CXCR4 scFvFcX33 or PBS was used as negative controls. The plate was processed with 50 µl HRP labeled streptavidin and color-developed with 100 µl TMB peroxidase substrate same as the procedure described above. The percentage of binding was calculated using the formulation: % = 100*(OD450 of labeled scFvFc + unlabeled scFvFc)/(OD450 of labeled scFvFc + PBS). Note that all ELISA samples were assayed in duplicates.

### Inhibiting Carbonic Anhydrase Activity of Soluble CAIX-ECD-Fc by Anti-CAIX scFvFc Antibodies

The procedure described online at http://www.worthington-biochem.com/CA/default.html was followed for the carbonic anhydrase activity assay. For blank determination: 4 ml chilled CO_2_ saturated water was added to 6.0 ml chilled 0.02 M Tris HCl buffer, pH 8.0 and the time (in seconds) required for the pH to drop from 8.3 to 6.3 was recorded as T_0_. 1 µg of CAIX-ECD-Fc in 100 µl of PBS was added to 6.0 ml chilled 0.02 M Tris HCl buffer, pH 8.0 immediately before adding of CO_2_ saturated water. The time needed for pH drop was set as T. The Unit activity of carbonic anhydrase was calculated according to the following formula: Units/mg = 2 × (T0-T)/(T×mg enzyme in reaction mixture). To determine the effects on CAIX activity, the anti-CAIX scFvFcs were mixed with 1 µg of CAIX-ECD-Fc at the molar ratio of Abs: enzyme  = 1∶1, 5∶1 or 25∶1 and then incubated at room temperature for 50 minutes. The mixture and 4 ml CO_2_ saturated water were added to 6.0 ml chilled 0.021 M Tris HCl buffer, pH 8.0 in a 50 ml Falcon tube, and the time required for the pH drop from 8.3 to 6.3 was recorded as T_Ab_. The small molecular carbonic anhydrase inhibitor, acetazolamide (ATZ, Sigma) and irrelevant scFvFcX33 were used as positive and negative controls, respectively. Units activity of carbonic anhydrase inhibited by scFvFcs was calculated according to the following formula: Units_Ab_/mg = 2×(T_0_−T_Ab_)/(T_Ab_×mg enzyme in reaction mixture) and the percentage of inhibition was calculated as: % of Inhibition  = 100×[1−(Units_Ab_/mg)/(Units/mg)].

### Antibody-Induced CAIX Internalization

#### General flow cytometry

293T-CAIX or 293T cells were incubated with individual anti-CAIX scFvFcs labeled with CypHer5E according to the manufacturer's instructions (GE Healthcare). The change in CypHer5E fluorescence, which is pH sensitive with minimal extra-cellular fluorescence in standard culture media and maximal intra-cellular fluorescence in acidic intra-cellular compartments such as the endosome, is used to monitor the effect of antibodies on internalization of the surface-expressed CAIX.

293T-CAIX cells were incubated with serum free DMEM medium for 4 hours and collected with 5 mM EDTA/PBS. 2.5×10^6^ cells were incubated with a saturating amount (2.5 µg) of CypHer5-labelled anti-CAIX scFvFcs in a 100 µl of PBC/0.5%BSA at 37°C or on ice for 15, 30, 45 or 60 minutes. Irrelevant scFvFc11A and scFvFcX48 as well as free CypHer5 dye were used as negative controls. YO-PRO-1 nucleic acid staining (Invitrogen) was used to monitor cell viability by incubating with cells for 20 min on ice. Unbound antibodies and stains were removed by washing the cells with PBS/0.5%BSA for three times. Cells were analyzed by general flow cytometry using FACScalibur (BD).

#### Quantitative imaging flow cytometry with ImageStream system (Amnis Corp, Seattle, WA) was used for colocalization studies

Upon staining with CypHer5-labelled anti-CAIX scFvFcs G37, G119, and G36, cells were washed, permeabilized and fixed with 100 µl of Perm/Fix solution for 20 min at RT. Cells were sequentially stained in 100 µl of Perm/Wash buffer containing 10 µg/ml FITC mouse anti-human EEA1 or PE-Cy5 mouse anti-human CD107a (LAMP1) for 20 minutes on ice. The cells were further washed twice with 1 ml of Perm/Wash buffer each, fixed cells with 1% paraformaldehyde, adjusted to 5×10^7^/ml (2.5×10^6^/50 µl) and transferred to 500 µl siliconized microcentrifuge tubes for analysis by ImageSream system. 5,000–15,000 event multispectral images were acquired for each sample on an ImageStream imaging flow cytometer (Amnis Corp, Seattle, WA). Single color control samples were used to create a compensation matrix that was applied to all files to correct for spectral crosstalk, and the resulting compensated image data was analyzed in the IDEAS software program (Amnis). Single cell events with intermediate area and high aspect ratio were gated to eliminate debris (low area) and multi-cellular events (large area, low aspect ratio) from further analysis. Bright punctate EEA1 or LAMP1 staining areas with a diameter of 3 microns or less were automatically counted using the spot count feature for EEA1+LAMP1 +CypHer+ and EEA1+LAMP1+CypHer- cells.

## Results

### Construction and Characterization of CAIX-PMPLs

**Figure 1:**
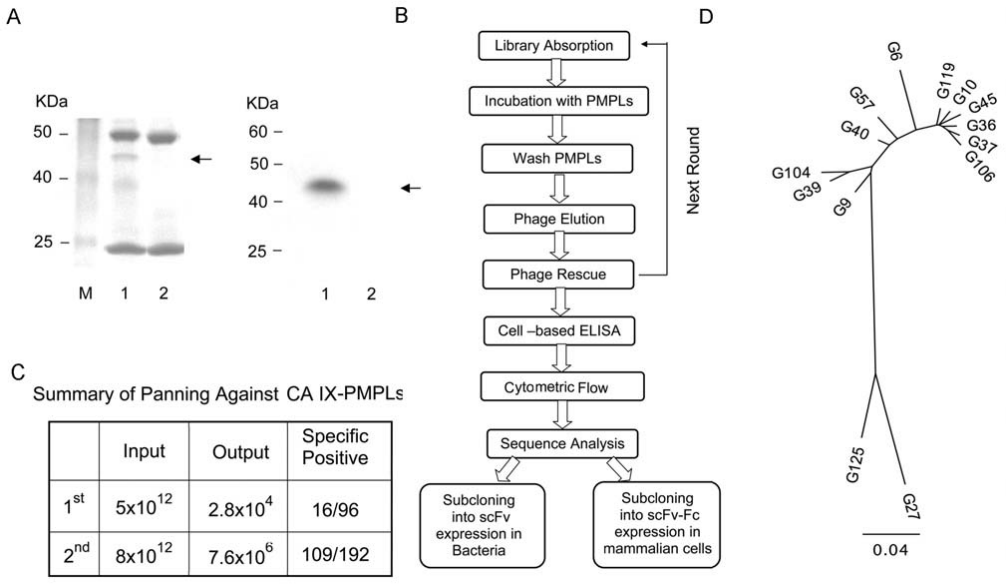
Characterization of CAIX paramagnetic proteoliposomes (CAIX-PMPLs). ***A (left)***, 3×10^7^ CAIX-PMPLs (lane 1) or M280 Dynal beads coated with 1D4 mAb only (lane 2) were treated with 2xSDS-buffer for 1 hr at 55°C followed by boiling for 5 min. The supernatant were used for SDS-PAGE analysis and proteins were visualized by Coomassie Blue staining. ***A (right)***, PMPLs were incubated with [^35^S]-methionine/cysteine labeled 293T-CAIX-C9 (lane 1) or 293T (lane 2) cell lysates. Purity of the PMPLs was determined by SDS-PAGE followed by autoradiography analysis. ***B.*** Schema of the antibody panning and selection process. ***C***. Summary of CAIX-PMPL panning results. ***D***. Phylogenetic tree of the fourteen (14) unique anti-CA IX antibodies based on translational alignment of nucleotide sequence comparison using Geneious (http://www.geneious.com/) software with PHYML tree plugin (http://atgc.lirmm.fr/phyml). Jukes-Cantor genetic distance model were utilized. Consensus tree option was selected with 100 bootstraps. The unit of measure (the scale bar) represents the number of amino acid substitutions per site.

Cell lysates from stably transfected 293T-CAIX cells were used to prepare CAIX-containing paramagnetic proteoliposomes (CAIX-PMPLs) [38], [39]. CAIX-PMPLs were pelleted, treated under reducing conditions, and the supernatant was subjected to SDS-PAGE analysis and visualized by Coomassie Blue staining. As shown in the left panel of Figure 1A, a circa 45 kDa mol wt band corresponding to CAIX is seen only in Lane 1, which was captured from cell lysate by the 1D4-coupled M280 beads. The two other protein bands detected in Lanes 1 and 2 correspond to the 1D4 mAb heavy chain (50 kDa) and light chain (25 kDa). Other cellular proteins were present at only trace levels. CAIX-PMPL purity was further checked by using cell lysates metabolically labeled with ^35^S-methionine/cysteine. The right panel of Figure 1A represents an autoradiogram that shows only one predominant band with the expected mol. wt. of CAIX was observed in PMPLs constructed with 293T-CAIX cell lysates (lane 1), while no band was detected from PMPLs formed with parental 293T cell lysates (lane 2).

### Isolation of CAIX-Specific Antibodies from a Phage Display Library

Two non-immune human scFv-phage display libraries containing 12 (Mehta I) and 15 (Mehta II) billion members were combined and used to directly isolate CAIX specific antibodies. The panning and selection processes are illustrated in Figure 1B and results summarized in Figure 1C. Sixteen out of 96 clones from the first round and 109 out of 192 clones from the second round of CAIX-PMPL panning specifically bound to 293T-CAIX cells as detected by cell-based ELISA and confirmed by flow cytometric analysis with phage antibodies (data not shown). DNA sequence analysis revealed a total of fourteen unique scFv clones (Table S1). There is a predominance of V_H_ germline V_H_3-23 usage that has recombined with five different JH segments to form the rearranged heavy chain genes. Likewise, the V_λ_ germline V1-40 gene was predominantly used in combination with J_λ_2-3 segments. A phylogenetic tree based on translational alignment of nucleotide sequence comparison [40] of these fourteen antibodies is presented in Figure 1D.

### Cell Surface Binding of Anti-CAIX scFvFcs Measured by Flow Cytometry

**Figure 2:**
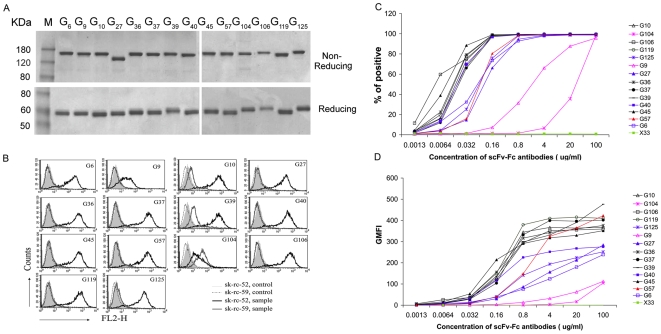
Characterization of selected anti-CAIX scFvFc antibodies. ***A***. Different anti-CAIX scFvFcs were expressed and purified from 293FT cells as discussed in Material and Methods. A total of 2 µg purified each anti-CAIX scFvFc were treated with 5XSDS-PAGE loading buffer (Pierce) in the presence (lower panel) and absence (upper panel) of reducing reagent DTT. The antibodies were separated by SDS- 10% Tris-Glycine PAGE gel electrophoresis and visualized by Coomassie Blue staining. ***B.*** Flow cytometric analysis of the anti-CAIX scFvFc binding activity. One microgram (1 µg) of each anti-CAIX scFvFc was incubated with 5×10^5^ sk-rc-52 (CAIX positive) or sk-rc-59 (CAIX negative) cells in 50 µl FACS buffer (1% BSA/PBS), followed by staining with PE-conjugated goat anti-human IgG. Binding activity of each antibody was examined by flow cytometric analysis and data is presented as overlapping histograms of the CAIX positive and negative cells as well as the secondary antibody only controls for each cell line. Similar findings were found in two independent experiments. ***C & D***. Dosage-dependent binding of the anti-CAIX scFvFcs to 293T-CAIX cells. Anti-CAIX scFvFcs or a control of irrelevant anti-CXCR4 scFvFc (X33) at the concentrations indicated on the X-axis were incubated with 5×10^5^ 293T-CAIX cells followed by staining with FITC conjugated goat anti-human IgG. Flow cytometric analysis was performed and binding activity of each antibody is presented as both percentage of positive cells (***C***) and absolute geometric mean fluorescence intensity (GMFI) (***D***). A summary of the calculated EC_50_ for half-maximal percent binding (***C***) and GMFI (***D***), along with maximal percent positive cells and GMFI reached, are presented in Table 1.

All fourteen anti-CAIX scFvs were subcloned, expressed in 293FT cells, and purified as scFvFc fusion proteins (Figure 2A). The binding activities of these bivalent proteins were further examined by flow cytometric analysis. The results demonstrated that 13 anti-CAIX scFvFcs could recognize the CAIX positive human renal clear cell carcinoma (RCC) sk-rc-52 cells, but not the CAIX negative RCC sk-rc-59 cells. G104 scFvFc bound to both cells, therefore, its binding was considered non-specific (Figure 2B).

The relative affinity of each anti-CAIX scFvFc to native CAIX molecules was determined by staining of the 293T-CAIX cells using serial dilutions of each scFvFc in flow cytometric saturation binding studies (Figure 2 C&D). The concentration of antibody to reach 50% of maximum percentage positive staining cells and respective geometric mean fluorescence intensity (GMFImax) are shown in Table 1. All scFvFcs reached ∼100% positive binding at relatively low concentrations compared to that needed to obtain GMFI saturation. The EC_50_ values for reaching 50% positive binding ranged from 0.05 nM for G106 to 24.8 nM for G9. Similarly, the EC_50_s for the half-maximal GMFI also showed a broad range from 1.2 nM for G45 to 310.5 nM for G9 (Figure 2C and 2D, Table 1). However, in contrast to the attainment of near complete (≥98%) saturable binding reached by all the antibodies, the plateaus reached by the different antibodies were not the same. Antibodies G10, G36, G37, G39, G45, G57, G106 and G119, reach a higher plateau (GMFI max between 352 and 476) than G6, G27, G40 and G125 that fall in an intermediate group (GMFI max between 239 and 284). In the case of G9, the antibody did not reach a plateau of GMFI at the highest concentration tested (100 µg/ml).

**Table 1:** Anti-CAIX antibody affinity as analyzed by flow cytometry and SPR.

scFvFc Ab	KD (nM)	Maximum GMFI	EC50 (nM) GMFImax	EC50 (nM) Bindingmax
**G119**	1.49	415.23	2.9	0.18
**G10**	1.62	367.8	2.9	0.21
**G37**	1.89	401.8	3.9	0.22
**G106**	3.20	359.54	3.4	0.05
**G36**	3.22	376.15	2.6	0.17
**G39**	3.43	476.23	5.1	0.20
**G57**	4.25	422.61	17.6	0.92
**G45**	12.50	351.5	1.2	0.11
**G40**	21.78	275.47	2.6	0.21
**G27**	25.12	283.94	30.3	1.10
**G6**	25.90	238.54	33.3	0.80
**G125**	40.32	252.1	6.4	0.87
**G9**	99.58	160.53	310.5	24.78
**Correlation between SPR KD & Flow cytometry**	***R*** **calculated**	**−0.849 (p<0.01)**	**0.908 (p<0.01)**	**0.904 (p<0.01)**

The EC_50_ values represent the concentrations of scFvFcs required to reach half-maximal geometric mean fluorescence intensity (MGFI) or circa 50% positive cell staining by flow cytometric analysis, respectively. K_D_ values determined by SPR were taken from Tables 2 and 3.

### Kinetic Analysis of Anti-CAIX scFvFc Binding by Surface Plasmon Resonance (SPR)

The binding characteristics of each anti-CAIX scFvFc were further analyzed by SPR through a capture technique, where purified anti-CAIX scFvFc was captured onto a BiaCore CM4 CHIP immobilized with anti-human IgG-Fc antibodies. The carboxy-terminal C9-tagged extracellular domain of CAIX (CAIX-ECD, amino acids 1-391) purified from 293FT cell culture supernatant was used as the antigen analyte. The fitted kinetic sensorgrams are shown in Figure S1. Non-specific binding to the reference anti-human Fc surface was not observed. The anti-CAIX scFvFcs were ranked based on their affinity (K_D_ value) to CAIX-ECD-C9 (Tables 2 and 3). Note that the eight highest affinity antibodies were also the eight antibodies that displayed the higher GMFImax plateau in Figure 2D (K_D_≤12.50 nM) while the five lower affinity antibodies (K_D_≥21 nM) reached only the intermediate GMFI max plateau or for G9 never reached the plateau (also refers to Table 1). There is significant correlation between the EC_50_ for GMFI or maximal percentage of binding and K_D_ as determined by BiaCore (Table 1).

**Table 2:** Kinetic and equilibrium dissociation constants for anti-CAIX scFv-Fc/CAIX-ECD-C9 interactions.

scFvFc Ab	KD (nM)	ka (M^−1^s^−1^) (×10^5^)	kd(s^−1^) (×10^−3^)
**G119**	1.49±0.30	5.80±1.29	0.84±0.04
**G10**	1.62±0.50	5.50±1.46	0.84±0.02
**G37**	1.89±0.51	3.92±1.23	0.70±0.06
**G106**	3.20±0.72	4.72±1.58	1.44±0.14
**G36**	3.22±0.66	5.10±3.15	1.54±0.67
**G39**	3.43±0.19	4.64±0.06	1.59±0.11
**G57**	4.25±0.06	3.37±0.06	1.43±0.01
**G45**	12.50±0.07	6.17±0.37	7.70±0.04
**G40**	21.78±4.41	3.52±1.49	7.35±1.69
**G27**	25.12±3.62	2.24±0.43	5.54±0.27

The kinetic constants of all CAIX antibodies except G6, G125 and G9 were obtained by global analysis using a 1∶1 langmuir binding model (*A+B* 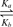 *AB*) using the T100 evaluation software. The values are the average of 2-3 different experiments.

**Table 3:** Kinetic and equilibrium dissociation constants for interactions of CAIX-ECD-C9 with scFv-Fc G6, G125, and G9 antibodies.

scFv-Fc Ab	KD (nM)	ka1 (M^−1^s^−1^) (×10^5^)	kd1 (s^−1^) (×10^−1^)	ka2 (s^−1^) (×10^−2^)	kd2 (s^−1^) (×10^−3^)
**G6**	25.90±2.38	3.75±0.27	2.02±0.03	1.11±0.01	0.56±0.05
**G125**	40.32±15.60	3.58±0.98	0.42±0.04	0.39±0.07	1.91±0.43
**G9**	99.58±23.36	2.24±0.49	2.37±0.90	1.63±0.06	1.71±0.07

When analyzed with the T100 evaluation software, G6, G125 and G9 curves indicate a more complex mechanism that will require further examination. However, for the purposes of the present study of comparative affinity and ranking of the antibodies, the data sets of G6, G125 and G9 were fitted to a 2-state model, *A+B* 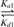 *AB* 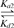 AB* and shown separately. The values are the average of 2–3 different experiments.

### Epitope Mapping Studies with CAIX Fusion Proteins

**Figure 3:**
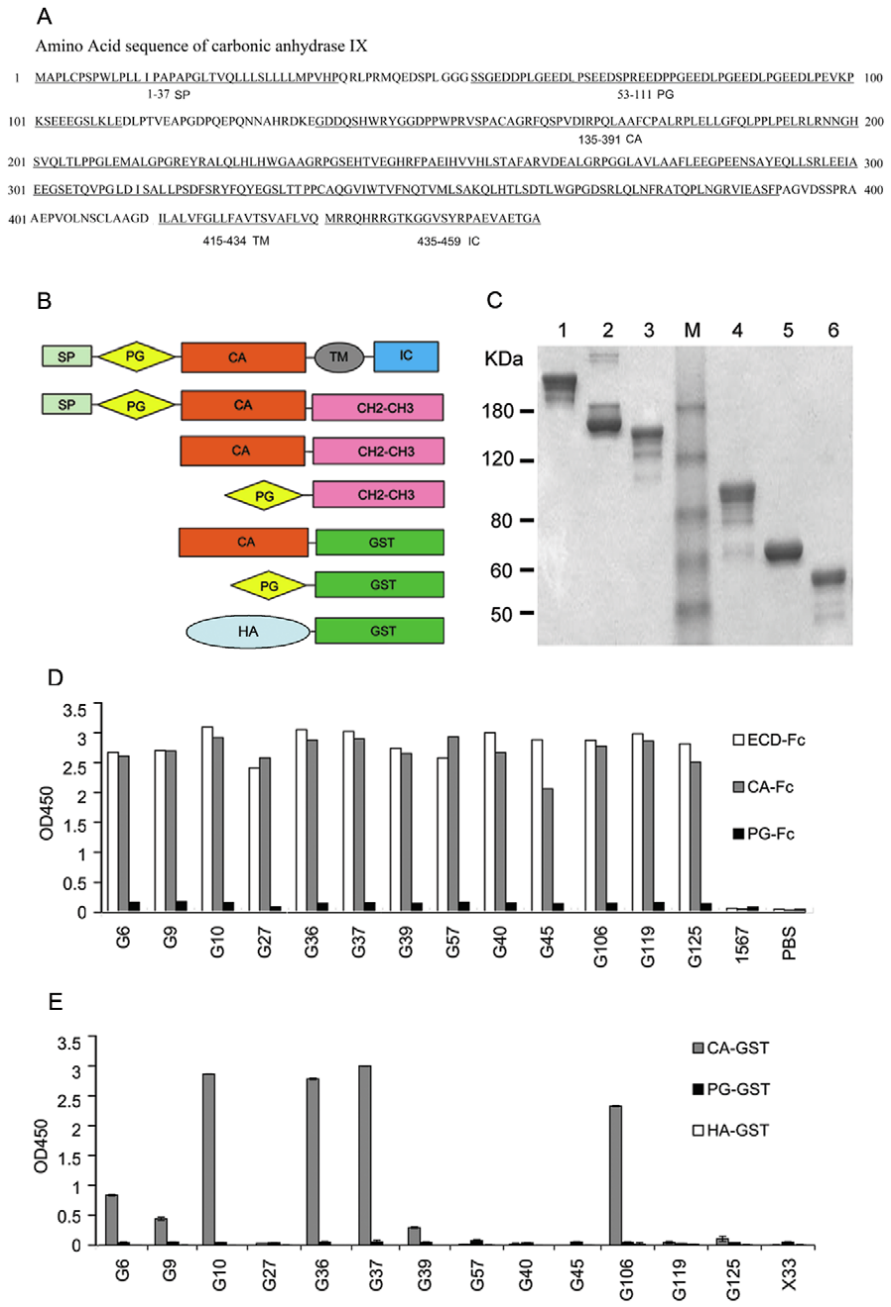
Binding specificity of the anti-CAIX antibodies as analyzed by domain mapping studies. ***A.*** Amino acid sequence of CAIX with amino acid locations of the different segment/domains as indicated. ***B***. Diagram of CAIX functional domains and fusion constructs. ***C***. Coomassie Blue staining of 2 µg purified CAIX-ECD-Fc (lane 1 & 4), CAIX-CA-Fc (lane 2 & 5), and CAIX-PG-Fc (lane 3 & 6) separated by SDS-PAGE under non-reducing (lanes 1-3) or reducing (lanes 4-6) conditions. ***D***. Binding of purified biotinylated CAIX-Fc constructs to the purified anti-CAIX scFvFcs analyzed by ELISA. ***E.*** Binding of purified anti-CAIX scFvFcs to the CAIX–GST fusion proteins analyzed by ELISA. For ***D*** and ***E***, average OD450 of each duplicated sample is presented. Similar results were obtained in two independent experiments. Details on the ELISA assays performed are provided in Experimental Procedures.

Purified CAIX-ECD-Fc, carbonic anhydrase domain (CA-Fc) and proteoglycan domain (PG-Fc) fusion proteins (Figure 3A–C) were used to map the CAIX epitopes recognized by the anti-CAIX antibodies in ELISA assays. Plates were coated with purified anti-CAIX or an irrelevant scFvFc antibody 1567 and incubated with equal molar concentrations of biotinylated CAIX-ECD-Fc, CAIX-CA-Fc or CAIX-PA-Fc. The data in Figure 3D indicates that all antibodies recognized the CA domain while none of the antibodies recognized the CAIX PG domain.

When reacted with CA-GST, PG-GST, or HA-GST (control) fusion proteins expressed and purified from bacteria (Figure 3B-C and data not shown), anti-CAIX scFvFc antibodies again did not recognize the CAIX PG domain coated on the plate (Figure 3E). The G10, G36, G37 and G106 had significant binding to the CA-GST (OD450 >2) while G6, G9, and G39 reacted with the CA-GST weakly. In contrast to the results in Figure 3D, G27, G40, G45, G57, G119 and G125 did not recognize the bacterially produced CAIX-CA-GST, suggesting that binding of these antibodies to the CA domain may be dependent upon unique protein folding of the CA domain and/or post-translational modification that are only present when produced in mammalian cells. The fact that CAIX has been shown recently to contain an intramolecular disulfide bridge, both *N*- and *O*-linked glycosylation [14], as well as tyrosine phosphorylation [41] supports our hypothesis. It should be noted that in addition to above biochemically characterized post-translational modifications, the NetPhos 2.0 Server software [42] (http://www.cbs.dtu.dk/services/) also predicts additional phosphorylation sites at tyrosine 289, threonine 333, and serine residues at 153, 162, 201, 237 and 287 sites with high confidence, along with threonine 205 predicted by the NetOGlyc 3.1 Server as a potential O-linked glycosylation site, all within the CAIX CA domain.

To understand further the nature of anti-CAIX scFvFcs binding within the CA domain, antibody cross-competition studies were performed. Specifically, purified anti-CAIX scFvFc proteins were biotinylated and flow cytometric competitive binding assays were set up to determine whether presence of an unlabeled anti-CAIX scFvFc could block the binding of any biotinylated scFvFcs to the CAIX expressed on cell surface, the latter detected using streptavidin-FITC. Diminished or reduced binding of the biotinylated scFvFc in the presence of non-labeled blocking antibody could indicate that antibodies have the same or overlapping binding sites (epitopes) or the existence of steric hindrance. Table S2 summarizes the results from this series of experiments. Antibodies are grouped based primarily on their amino acid sequence homology (represented by the same color shade of the antibody names) with the highest affinity mAb G119 at the top left and descending affinities either down the columns or moving to the right along each row. The “+” and “-” signs in Table S2 represent the percentage of biotinylated antibody binding remaining. In general, the highest affinity antibodies, as expected, show the greatest breadth of cross-competition. It appears that G119, G10, G37, G106, G36, G45, G39, G57, G40 and G6 share similar characteristics in that they bind to the same or overlapping epitopes in the CA region as they are capable of blocking or interfering all other antibodies binding to the CAIX ECD domain and the degree of blocking correlates strongly with their binding affinities (K_D_). Although the binding affinity of G27, G125, and G9 ranked the lowest among all the anti-CAIX antibodies selected (Tables 2 and 3), their binding could not be blocked completely by any other antibodies. The data indicate that G27, G125 and G9 have unique epitopes: while G9 and G125 may recognize same binding site based on their blocking patterns, G27 and G125 do not necessarily share the same epitope. This is evidenced by the fact that they do not block each other's binding and exhibit different blocking patterns to other antibodies. Finally, it should be noted that G119 only exhibited 20–40% self-blocking. We believe this was most likely a result of the experimental procedure employed since the cross-competition assay was performed in a mixture of biotin-labeled and unlabeled antibody at 1∶1 ratio in the absence of preincubation of CAIX-coated plates with unlabeled antibodies in excess. Under these conditions and considering the high affinity (slow off rate) of G119, it is not surprising to detect biotin-labeled G119 binding to CAIX even in the presence of unlabeled G119.

### Inhibition of Carbonic Anhydrase Activity by Anti-CAIX Antibodies

**Figure 4:**
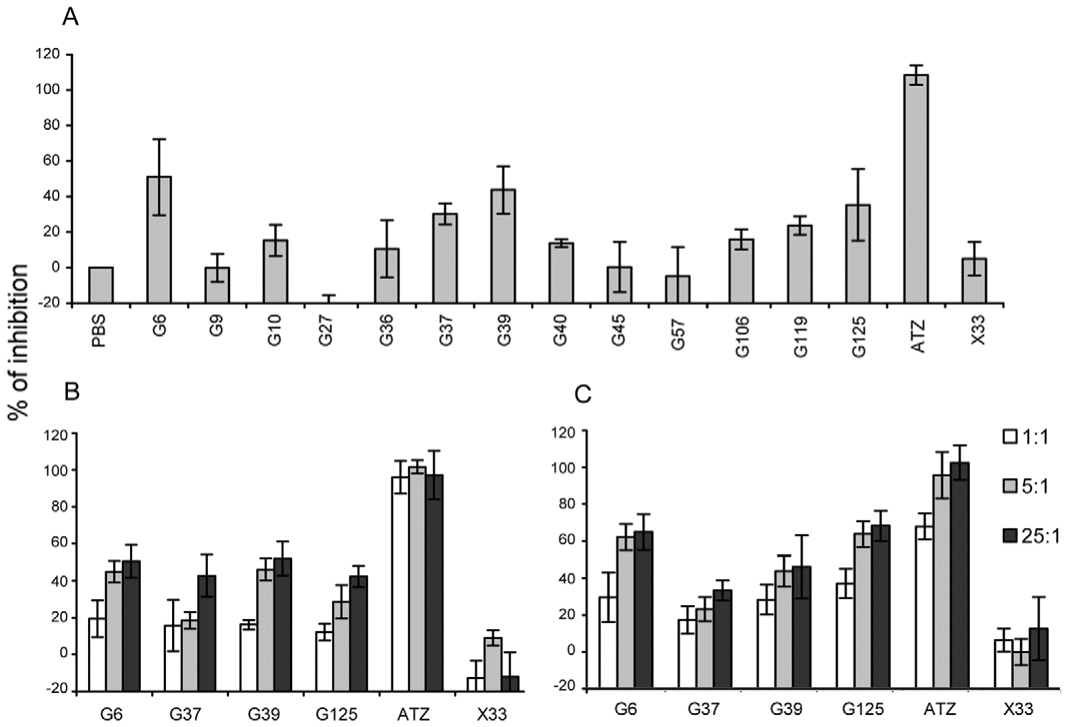
Inhibition of carbonic anhydrase activity of CAIX by the anti-CAIX scFvFc antibodies. The ability of the anti-CAIX scFvFc antibodies to inhibit the reaction CO_2_+H_2_O → H_2_CO_3_ catalyzed by CAIX (CAIX) was assessed using the electrometric assay as described in Experimental Procedures. Different molar ratio of antibody to CAIX enzyme was tested. The anti-CXCR4 scFvFc X33 and carbonic anhydrase inhibitor acetazolamide (AZT) were used as negative and positive control, respectively. Percent inhibition of CAIX enzymatic activity was calculated per formula discussed in the Experimental Procedures. ***A***. Inhibition of CAIX enzymatic activity by anti-CAIX-scFvFc at molar ratio 25∶1. ***B***. Inhibition of CAIX enzymatic activity by selected anti-CAIX-scFvFc at molar ratio 1∶1, 5∶1, and 25∶1. ***C***. Inhibition of CAIX enzymatic activity by selected anti-CAIX-scFv antibodies at molar ratio 1∶1, 5∶1, and 25∶1. Values represent an average of three different experiments.

Carbonic anhydrases (CA) catalyze the reaction: CO_2_ + H_2_O ↔ H_2_CO_3_. Since the anti-CAIX antibodies are epitope mapped to the CA domain, their abilities to block the carbonic anhydrase enzymatic activity of CAIX were tested. At a molar ratio of 25:1 and compared with svFvFcX33 negative control, G6, G37, G39 and G125 scFvFcs showed different degrees of inhibition on the CAIX-CA-Fc enzymatic activity (Figure 4A). Their inhibitory activities appear to be specific in a dose-sensative manner (Figure 4B). The monovalent scFv form of the G6 and G125 are as effective, if not more, in blocking carbonic anhydrase activity than their bivalent scFvFc counterparts (Figure 4C), presumably resulted from easier access of the catalytic site by a smaller antibody molecule.

### Flow Cytometric and Imaging Studies of Antibody Induced CAIX Internalization

**Figure 5:**
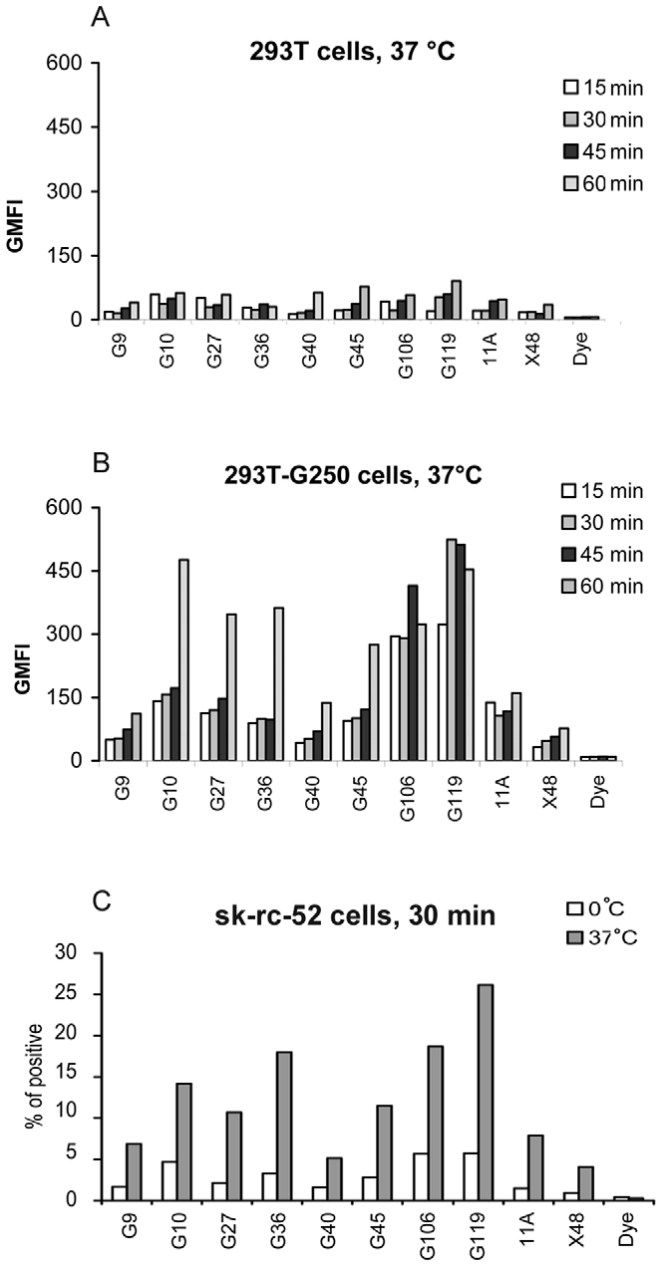
Induction of CAIX internalization from cell surface by anti-CAIX antibodies. CypHer5E-labeled anti-CAIX scFvFc antibodies were tested for their abilities to induce CAIX internalization by flow cytometric analysis. The 293T cells (***A***) or 293T-CAIX cells (***B***) were incubated at 37°C with 2.5 µg/100 µl CypHer5E-labeled anti-CAIX scFvFc antibodies for 15, 30, 45, or 60 minutes. Two irrelevant scFvFc antibodies, 11A and X48, as well as CypHer5E dye were used as negative controls. ***C*** represents a similar experiment where CAIX internalization in sk-rc-52 (CAIX positive) cells were analyzed and compared at 37°C and 0°C. Fluorescence intensity of cells was analyzed using FACS Calibur and GMFI value (***A*** & ***B***) or percent of CypHer5E positive cells (***C***) were presented in corresponding panels.

For the initial screening, 293T-CAIX or 293T cells were incubated with saturating amounts of CypHer5E labeled-anti-CAIX scFvFcs at 4°C or 37°C for 60 minutes and antibodies with appreciable amounts of CypHer5E fluorescence, an indication for internalization, were chosen for further time-course study (data not shown). As shown in Figures 5A and 5B, as compared with the negative control antibodies 11A and X48, G10, G27, G36, G45, G106, and G119 were found to induce CAIX internalization only in 293T-CAIX cells. This result was also confirmed with internalization studies using CAIX positive RCC cells sk-rc-52 at 37°C or on ice (Figure 5C).

**Figure 6:**
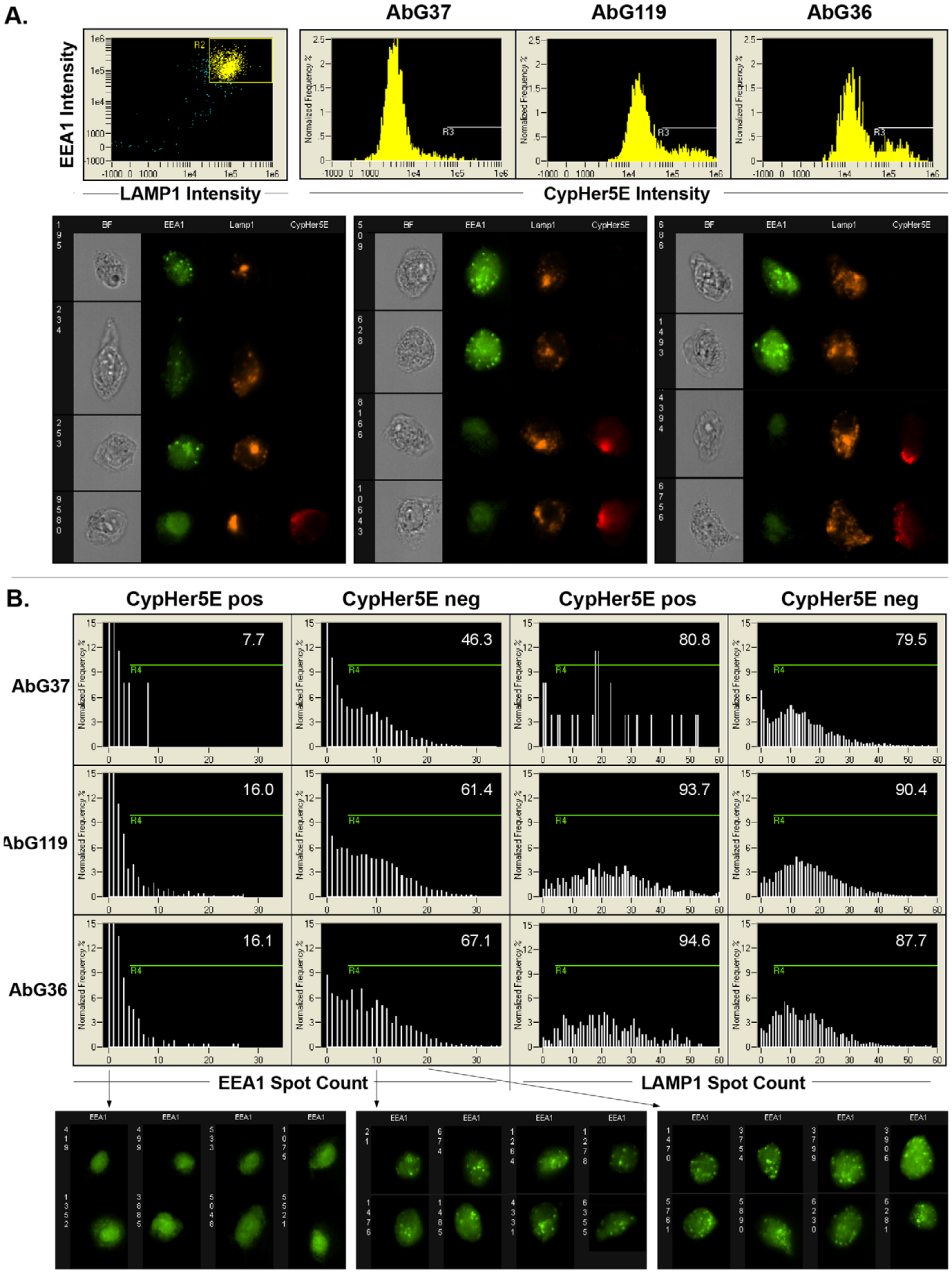
Antibody-induced CAIX internalization as analyzed by ImageStream. ***A.*** Single cells expressing EEA1 and Lamp1 were gated (R2), and the CypHer5E intensity was plotted for cells incubated with anti-CAIX G37, 119, or 36 (left, center, and right plots), with the relative percentage of CypHer5E+ events (R3) displayed in the upper right of each plot. Representative Brightfield, EEA1 (green), LAMP1 (orange), and CypHer5E (red) cell images from the three antibody treatment groups are shown below in the same order. ***B.*** The number of EEA1 or LAMP1 small punctate staining regions is plotted for CypHer5E+ (R3) or CypHer5E- cells from the indicated antibody treatment groups. The percentage of cells with greater than four spots (R4) is displayed in the upper right corner of each plot. Representative EEA1 images from the AbG36 sample of cells with zero (left), ten (center), or twenty EEA1 spots (right) are shown below the plots.

To determine the intracellular trafficking pattern of the internalized antibody-CAIX complexes, anti-CAIX scFvFc G36 and G119 were selected for co-localization studies of CypHer5E-labeled anti-CAIX antibodies to endosomes (stained with FITC anti-EEA1) or lysosomes (stained with PE anti-LAMP1) using quantitative imaging flow cytometry (Figure 6). Antibody G37 that did not induce CAIX internalization as analyzed by regular flow cytometry (data not shown) was used as a negative control. Figure 6A shows that once again, compared to the cells stained with G37 (0.9%), a significant percentage of 293T-CAIX cells incubated with G36 (16.8%) and G119 (19.2%) were CypHer5E positive. Representative cell images from CypHer5E positive and negative populations are also shown in Figure 6A. Although co-localization of CypHer5E staining with endosomal EEA1 or lysosomal LAMP1 markers were not visually observed within CypHer5E+ cells incubated with scFvFc G36, 119, or 37, a significant decrease in number of EEA1 spots in CypHer5E+ but not CypHer5E- cells was noted. This loss in EEA1 texture in CypHer5E positive cells was quantified and confirmed using the spot count feature of the IDEAS image analysis software by Amnis, which automatically counts bright punctate staining areas less than 3 microns in diameter. As shown in Figure 6B, percentage of cells containing more than 5 EEA+ spots was greatly reduced among CyeHer5E+ compared to the CyeHer5E- cells while no significant differences were observed regarding the LAMP1 spot count; indicating that internalization of antibody and CAIX complexes into endosomes may have caused quenching of the EEA-FITC signal.

## Discussion

Epitopes expressed on the cell surface of tumor cells are superior targets for anti-cancer immune therapy since, unlike intracellular antigens; they are accessible to circulating antibodies *in vivo*. In the last few years, human monoclonal antibodies (mAbs) have become a well-tolerated and preferred treatment option for an increasing number of cancers as they do not induce HAMA (human anti-mouse-antibody) or HACA (human anti-chimeric-antibody) responses when used in clinical applications. As accumulating evidence indicates that CAIX is a very promising target for tumor immune therapy and diagnosis, there is a great need for discovery of additional anti-CAIX antibodies, especially antibodies of human origin, to fulfill the need for various clinical applications.

In this study, using PMPL incorporated with CAIX in its native conformation, we selected and characterized 14 human antibodies from a large human scFv antibody library. The antibodies with diverse functions were isolated very early during the selection (Figure 1C), again demonstrating that PMPL is a powerful tool for isolation of human antibodies against self-antigens when used in combination with the large naïve human scFv phage display library [38], [39]. Sequence analysis indicates that most of the antibodies belong to the V_H_3-23 of H chain (13 Abs) and V1-40 of λ light chain (12 Abs) families. Other germlines represented include V_H_3-30 for G27 and V_λ_3-21 for G27 and G125. As expected, significant sequence diversity resides within the CDR, especially CDR3 of both H and L chains. Thirteen antibodies interact with overlapping or different epitopes within the CA domain and seven with measured affinity binding constant (KD) between 1.5 nM to 4.3 nM. At least four antibodies are capable of inhibiting the CA activity and six induce surface CAIX internalization efficiently (see Table 4 for summary).

**Table 4:** Summary on properties of the newly discovered human anti-CAIX antibodies.

CAIX Abs	Affinity (KD) (nM)	Mapping* (CA-GST) (OD450)	Cross-competition Grouping	% CA Inhibition by anti-CAIX scFvFc	Internalization
**G119**	1.49	−	1	25	+++***
**G10**	1.62	3	1	15	+
**G37**	1.89	3	1	**40** **	−***
**G106**	3.20	2.4	1	20	++
**G36**	3.22	3	1	10	++***
**G45**	12.50	−	1	−	+
**G39**	3.43	0.25	1	**50** **	−
**G57**	4.25	−	1	−	−
**G40**	21.78	−	1a	15	−
**G6**	25.90	0.9	1a	**50** **	−
**G27**	25.12	−	2	−	+
**G125**	40.32	−	3	**40** **	−
**G9**	99.58	0.4	3	−	−

*All anti-CAIX antibodies recognize the CA domain but only a selected group interacts with bacterially produced CA-GST (Figure 3D &E).

**Percentage of carbonic anhydrase inhibition by CAIX antibodies was estimated from Figure 4B. The rest data are from Figure 4A.

***Internalization measured by both flow cytometry and ImageStream. + and − denotes degree of internalization as measured by regular flow cytometry (Figure 5).

To date, antibodies against human CAIX molecules have been generated by traditional hybridoma technology through fusion of myeloma cells with the splenocytes from mice immunized with primary RCC lesions [16], Hela cells [13], and more recently from CAIX-deficient mice immunized with NIH3T3-CAIX cells with or without purified GST-CA protein boosts [37]. The generated CAIX antibodies primarily reacted with the PG domain unless the immunization scheme contained a boost injection of purified recombinant CA domain, presumably due to immunodominance of the N-terminal located and highly acidic PG domain [43]. Armed with CAIX-PMPL and phage display technology, the human anti-CAIX antibodies isolated in vitro avoided immunudominance of the PG domain and all recognized epitopes in CA region. Based on cross competition studies, these antibodies may recognize ≥3 different epitopes. In addition, four antibodies G6, G37, G39, and G125, clearly demonstrated their abilities of inhibiting carbonic anhydrase activity of the CAIX-PMPL, a novel biological activity that has not been previously described for CAIX antibodies. Since the enzymatic activity is believed to contribute to CAIX ability to promote tumor growth/survival [22], [23] and a carbonic anhydrase inhibitor has been reported to suppress invasion of renal cancer cells in vitro [44], the newly discovered CA-inhibitory antibodies can be tested for their anti-RCC activity in vivo, which could provide a different mechanism for RCC immune therapy.

Six CAIX antibodies discovered exhibited different degrees of abilities to induce internalization of surface-expressed CAIX as judged by pH sensitive CypHer 5E labeled antibodies using flow cytometry (Figure 5). In addition, G119 and G36 induced CAIX internalization to endosomes was confirmed by imaging (Figure 6). These antibodies could be conjugated with a toxin to achieve targeted tumor killing or fused with a nucleic acid-binding moiety for target therapeutic gene delivery [45], [46]. On a separate front, antibodies specific to tumor surface markers, based on its internalization function, could be used for molecular imaging with different radioisotope labels and aid in diagnostic of tumor type and choice of treatment plans [35]. It should be pointed out that antibodies G45, G119, G10, G37, G36, and G106, although belong to the same cluster in the genetic tree and have identical CDRs in their V_H_ regions (Figure 1D and Table S1), do not necessarily possess same characteristics. For example, compared with the other 5 antibodies in this group, G45 has a low affinity constant (KD = 12.5 nM) and is not able to inhibit CA activity or bind to bacterially produced CA-GST fusion protein. G37, while having similar properties in most criteria as other Abs in this group, failed to induce CAIX internalization (Figure 6 and Table 4). These results indicate that the amino acid sequence changes in V_L_ CDRs are the likely contributing factors for the functional difference. This is especially notable in the case of G36 and G37, where the only significant difference is a R to S change in V_L_ CDR3 (Table S1), yet G36 internalizes whereas G37 does not. To our knowledge, this unique property of antibodies has not been previously reported. Additional studies on specific residues in V_L_ CDRs, especially X-ray crystallographic and/or mutational studies, could further our understanding of the CAIX structural/function relationship, the critical contact residues of the antibody, as well as rational design of a better therapeutic anti-CAIX antibody.

Many mechanisms have been proposed for the ability of antibodies to mediate their anti-tumor effects *in vivo*. For example, engagement of the antibody Fc domain with effecter cell FcγRs leads to antibody-dependent cell-mediated cytotoxicity (ADCC). Some (antagonist or inhibitory) antibodies can block the signaling on tumor cells and in this way may act synergistically with immune effector responses by rendering the tumor cells more susceptible to immune effecter cell triggered apoptosis or lytic cell death [47]. Another way that antibodies can be utilized is through the construction and functional expression of chimeric-immune receptors or “T-bodies” on T-lymphocytes otherwise known as “designer T-cells” [48], [49]. T-lymphocytes grafted with a chimeric receptor have the combined advantages of MHC-independence and antibody-based antigen binding with efficient T-cell activation upon specific binding to the receptor ligand. On the other hand, single-chain antibody-based bispecific T cell engagers (BiTE), which are capable of engaging cytotoxic T cells for lysis of tumor cells, have recently been shown to have therapeutic potential for treatment of non-Hodgkin's lymphoma in a clinical setting [50]. It worth noting that as CAIX expression, which is trans-activated by hypoxia inducible factor-1α [51], has been reported in increasing cases of other cancers, such as lung, cervical, ovarian, esophageal, breast, prostate, bladder, and head and neck squamous cell carcinomas [52]–[54], the human CAIX antibodies discovered in this study will become very valuable reagents and be evaluated for their ability against different types of cancer through not only each above-mentioned mechanism but also their newly discovered carbonic anhydrase inhibitory effects.

## Supporting Information

Figure S1 Characterization of anti-G250 scFv-Fcs based on their kinetic interactions with the antigen, G250-ECD-C9. The antibody/antigen interactions were studied by SPR via a capture technique, with anti-human Fc as ligand and purified CA IX-ECD-C9 as analylyate using Biacore T100. Sensograms shown are double referenced (reference surface and blank-buffer subtracted). Five different concentrations of the analyte diluted serially from 50, 100 or 400 nM were used for different antibodies (50 nM- G119, G10, G37; 100 nM- G106, G36, G39, G57, G45, G40, G27; 400 nM- G6, G125, G9). The kinetic constants were obtained by global analysis using a 1∶1 Langmuir binding model (shown in black) using the T100 Evaluation software for comparative analysis of the antibodies. G6, G125 and G9 showed better fitting to a two-state model. The kinetic constants shown in Table 3 were the average of 2–3 assays.
(0.11 MB PDF)

Table S1 Sequence analysis of CAIX-specific antibodies including V and J germline gene usage.
(0.08 MB PDF)

Table S2 Binding site analysis through cross-competition studies with different anti-CAIX antibodies.
(0.66 MB PDF)

Text S1 Supplemental Materials and Methods.
(0.05 MB DOC)
