# Supplementary figures and images for: Unique Biological Properties of Catalytic Domain Directed Human Anti-CAIX Antibodies Discovered through Phage-Display Technology
Source: PLoS One. 2010 Mar 10;5(3):e9625. doi: 10.1371/journal.pone.0009625 (PMC2835754; doi:10.1371/journal.pone.0009625)

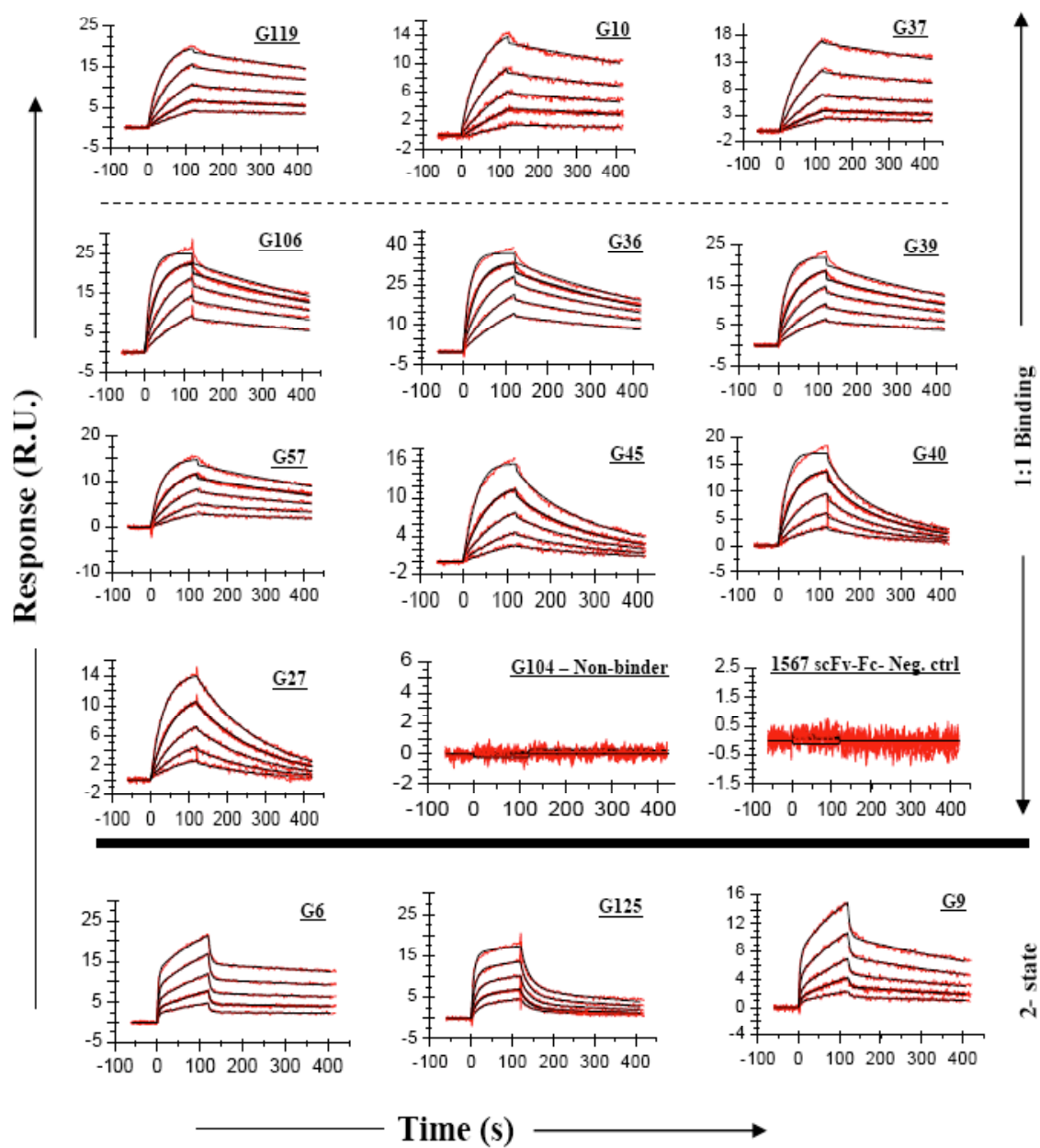

Supplement: Figure S1 — Characterization of anti-G250 scFv-Fcs based on their kinetic interactions with the antigen, G250-ECD-C9. The antibody/antigen interactions were studied by SPR via a capture technique, with anti-human Fc as ligand and purified CA IX-ECD-C9 as analylyate using Biacore T100. Sensograms shown are double referenced (reference surface and blank-buffer subtracted). Five different concentrations of the analyte diluted serially from 50, 100 or 400 nM were used for different antibodies (50 nM- G119, G10, G37; 100 nM- G106, G36, G39, G57, G45, G40, G27; 400 nM- G6, G125, G9). The kinetic constants were obtained by global analysis using a 1∶1 Langmuir binding model (shown in black) using the T100 Evaluation software for comparative analysis of the antibodies. G6, G125 and G9 showed better fitting to a two-state model. The kinetic constants shown in Table 3 were the average of 2–3 assays. (0.11 MB PDF) [file pone.0009625.s001.pdf]
