# Supplementary material for: Unique Biological Properties of Catalytic Domain Directed Human Anti-CAIX Antibodies Discovered through Phage-Display Technology
Source: PLoS One. 2010 Mar 10;5(3):e9625. doi: 10.1371/journal.pone.0009625 (PMC2835754; doi:10.1371/journal.pone.0009625)
Supplement: Table S1 — Sequence analysis of CAIX-specific antibodies including V and J germline gene usage. (0.08 MB PDF) [file pone.0009625.s002.pdf]

Table S1. Sequence analysis of CAIX-specific antibodies including V and J germline gene usage

A. Heavy Chain Variable Region

	10 20 30		40	50 a 60	70 80 abc 90	100 abcde	110	Germline VH gene	Germline JH gene	V&J Mutation
C	QVQLVQSGGGLVQPGGSLRLSCAASGFTFS	SYAMS	WVRQAPGKGLEWVS	AISGSGGXTYYADSVKG	RFTISRDNSKNTLYLQMNSLRAEDTAVYYCAN	N-GN--YRGAFDI	WGQGTLVTVSS			
G119	...........................P..	.....	....	...AN..T.........	....	....	.....M.....	V_H3-23	J_H3	7
G10	...........................P..	.....	....	...AN..T.........	....	....	.....M.....	V_H3-23	J_H3	7
G45	...........................P..	.....	....	...AN..T.........	....	....	.....M.....	V_H3-23	J_H3	7
G37	..........V................P..	.....	....	...AN..T.........	....	....	.....M.....	V_H3-23	J_H3	8
G106	E.............................	.....	....	...AN..T.........	....	....	.....T.....	V_H3-23	J_H3	5
G36	E.........V................P..	.....	....	...AN..T.........	....	....	.....M.....	V_H3-23	J_H3	7
G57	....	.....	....	......VS.........	...............................K	YCSSTSCYRGM.V	..K........	V_H3-23	J_H6	3
G40	....	.....	....	.......S.........	...............................T	Y..D..-Y.SL.Y	....	V_H3-23	J_H4	3
G6	.........................E...G	T...T	....	.V.....S.........	.........R.....................R	GPV-..L.YG...	.....M.....	V_H3-23	J_H3	10
G104	....QE........................	I....	....	.......G..H......	...............................K	F.SA...S.-Y.L	....	V_H3-23	J_H5	5
G39	....QE........................	.....	....	.......S.........	...............................K	I..R...SSSLGY	....	V_H3-23	J_H4	2
G9	....QE........................	.....	....	.......S.........	...............................R	SHSS..--.G..Y	....	V_H3-23	J_H4	2
G27	....KE....V....T..............	N...T	.............G	L..YD.SV.H.T.....	........A..S......T...D........R	-..S..--.YQEH	....	V_H3-30	J_H4	15
G125	E....E....V....R..............	..G.H	....	.......S.........	........A......................R	-.—AAVT.G..P	....	V_H3-23	J_H1	6
	FW1	CDR1	FW2	CDR2	FW3	CDR3	FW4			

B. Light Chain Variable Region

	10 20	abc 30	40	50	60 70 80	90 abc	100	Germline Vλ gene	Germline Jλ gene	V&J Mutation
C	QSVLTQPPSVSGAPGQRVTISC	TGSSSNIGAGYDVH	WYQQLPGTAPKLLIY	GNXNRPS	GVPDRFSGSKSGTSASLAITGLQAEDEADYYC	QSYDSSLXAW-V	FGGGTKLTVL			
G119	....	....	....	..T....	...................I....D.......	.....T.RV..M	....	V1-40	J_L3	6
G10	....	....	....	..S....	............S...............H...	....R..S-...	....	V1-40	J_L3	4
G45	.................I....	..T...........	.......A..RV...	..N....	.......................S........	....K..S-...	....	V1-40	J_L3	9
G37	....	....	...H...........	..S....	...........................T..F.	.......S....	......V...	V1-40	J_L2/ J_L3^a	4
G106	....	..........F...	..........R....	..N....	...........................T..F.	.......S....	......V...	V1-40	J_L2/ J_L3^a	6
G36	....	..........F...	....	..T....	...........................T....	.....R.S....	....	V1-40	J_L3	5
G57	....	....	....	A.N....	....	.......R....	.......A..	V1-40	J_L3	4
G40	....	....	....	A.N....	....	.......R....	.......A..	V1-40	J_L3	4
G6	.................I....	...R.....D....	....	A.N....	...G...A...........S............	.......R....	.......A..	V1-40	J_L3	10
G104	....	........R..N..	....	D.T....	...A.......A.....T......D.......	.....G.R-...	........L.	V1-40	J_L3	11
G39	....	........R..N..	....	D.T....	...A.......A............D.......	.....G.R-...	........L.	V1-40	J_L3	10
G9	....	........R..N..	....	..T....	...........................G....	.......S....	....	V1-40	J_L3	5
G27	LP.........V....TAR.T.	G.--N...SK-S..	....K..Q..V.V..	YDSD...	.I.E.....N..NT.T.T.SRVE.G.......	.VW...SDHHV.	....	V3-21	J_L2/ J_L3^a	4
G125	.PG........V....TAR.T.	G.--D...RK-S..	....R..Q..I.V.R	DDRD...	.I.E.....S.VNT.T.I.SRVE.G.......	.VW...SKHY..	..P...V.A.	V3-21	J_L1	14
	FW1	CDR1	FW2	CDR2	FW3	CDR3	FW4			

"." = Matches exactly to consensus

"-" = filler space

"X" = in consensus is various AA

"C" = Consensus

The linker in G40, G57, G119 all have one AA change

"V&J Mutation" = # of V&J mutations from the germline

[a] These genes are equally homologous to both germlines
