# Supplementary material for: Unique Biological Properties of Catalytic Domain Directed Human Anti-CAIX Antibodies Discovered through Phage-Display Technology
Source: PLoS One. 2010 Mar 10;5(3):e9625. doi: 10.1371/journal.pone.0009625 (PMC2835754; doi:10.1371/journal.pone.0009625)
Supplement: Table S2 — Binding site analysis through cross-competition studies with different anti-CAIX antibodies. (0.66 MB PDF) [file pone.0009625.s003.pdf]

Table S2: Binding site analysis through cross-competition studies with different anti-CAIX antibodies.

Unlabeled Competing Antibodies (columns); **Biotinylated Antibodies** (rows)

	G119	G10	G37	G106	G36	G45*	G39	G57	G40	G6	G27	G125	G9	X33	PBS
**G119**	+++	+++	+++	++++	++++	++++	++++	++++	++++	++++	++++	++++	++++	++++	++++
**G10**	-	-	-	++	++	++	++++	++++	++++	++++	++++	++++	++++	++++	++++
**G37**	-	-	-	+	-	+	-	+	+++	++++	++++	++++	++++	++++	++++
**G106**	-	-	-	-	-	-	++	++	++	+++	++++	++++	++++	++++	++++
**G36**	-	-	-	-	-	-	-	-	-	++	+	-	++++	++++	++++
**G45***	-	-	-	-	-	-	-	-	-	++	+	-	++++	++++	++++
**G39**	-	-	-	-	-	-	-	+	+	++++	+++	++	++++	++++	++++
**G57**	-	-	-	-	-	-	-	-	-	+	++	-	++	++++	++++
**G40**	-	+	+	+	-	-	+	+	-	+	+++	+	+	++++	++++
**G6**	+	+	-	-	-	-	+	+	+	+	++	+	+	+++	++++
**G27**	+	+	+	+	+	+	++	++	++	++	+	+++	+++	++++	++++
**G125**	+	+	++	+	+	++	++	++	++	++	+++	+	+	++++	++++
**G9**	+	+	+	+	++	+	+	++	+	+	++++	+	+	+++	++++

Antibody names with the same color-fill are on the same branch of the genetic tree (see Supplemental Table S1).
*The table is arranged according to the ranking of antibody affinity with the highest from top to bottom and left to right except that G45 is lower than that of G39 and G57.
Formula: % binding remaining = 100*(OD450 of biotinylated protein plus unlabeld protein) / (OD450 of biotinylated protein plus PBS)
Ranking / shade designation for percent of Biotin/Streptavidin binding remaining

>80%: ++++	60-80%: +++	40-60%: ++	20-40%: +	< 20: -
