# Supplementary material for: Unique Biological Properties of Catalytic Domain Directed Human Anti-CAIX Antibodies Discovered through Phage-Display Technology
Source: PLoS One. 2010 Mar 10;5(3):e9625. doi: 10.1371/journal.pone.0009625 (PMC2835754; doi:10.1371/journal.pone.0009625)
Supplement: Text S1 — Supplemental Materials and Methods. (0.05 MB DOC) [file pone.0009625.s004.doc]

**Text S1**

**Unique Biological Properties of Catalytic Domain Directed Human Anti-CAIX Antibodies Discovered Through Phage-Display Technology**

**Chen Xu^1, 3^, Agnes Lo^1, 3^, Anu Yammanuru^1^, Aimee St. Clair Tallarico^1^, Kristin Brady^1^, Aki Murakami^1^, Natasha Barteneva^2, 4^, Quan Zhu^1, 3^, and Wayne A. Marasco^1, 3^**

From Department of Cancer Immunology & AIDS, Dana-Farber Cancer Institute^1^, Immune Disease Institute and Program in Cellular and Molecular Medicine, Children's Hospital^2^,

Department of Medicine^3^ and Department of Pathology^4^, Harvard Medical School, Boston, MA 02115

*Antibodies and reagents -* The sources for antibodies and reagents are: The 1D4 mAb reacting with a C9 peptide - the University of British Columbia (Vancouver, Canada). FITC-goat anti-human IgG (Sigma). Purified non-labeled and horseradish peroxidase (HRP)-conjugated mouse IgG against M13 - Amersham Pharmacia Biotech Inc. Biotinylation kit and streptavidin-HRP were from Pierce. Other reagents included *Escherichia coli* TG1 and helper phage VCS M13 (Stratagene, La Jolla, CA, USA); G418 (Cellgro, Herndon, Va.); TMB substrate and stop solution (KPL, Gaithersburg, MD).

*Molecular Cloning and expression of CAIX proteins and anti-CAIX antibodies-*

*- Cloning of full length CAIX cDNA into mammalian expression vector and establishing CAIX expressing stable cell line.* The gene of full length CAIX was obtained by RT-PCR from mRNA of human cervical cancer Hela cells (ATCC) and cloned into pcDNA3.1-CD5L-C9 vector at NheI/BspE I sites by using primers 5’- TCT AGC TAG CCG CCA CCa tgg ctc ccc tgt gc –3’ (forward) and 5’-AAG GTC CGG Agg ctc cag tct cgg cta cct-3’ (reverse). The resulting plasmid, pcDNA3.1-CD5L-CAIX-C9 DNA, which contains a N-terminal CD5 leader peptide and C-terminal C9 tag (TETSQVAPA-corresponding to a region of human rhodopsin) was used to transfect 293T cells with Polyfect transfect reagent (Qiagen) according to the instruction. Stably transfected CAIX-expressing cell line 293T-CAIX was maintained in complete DMEM containing 500µg/ml G418.

*- Construction and expression of soluble CAIX extracellular domain fusion proteins*. Construction and expression CAIX-ECD-C9, CAIX-ECD-Fc, PG-Fc and CA-Fc fusion proteins: The DNA fragment encoding CAIX extracellular domain (ECD domain, amino acid 38-397) was amplified by PCR using pcDNA3.1-CD5L-CAIX-C9 as a template and cloned into pcDNA3.1-hinge-stuffer vector via Sfi I/Not I sites, where the CAIX ECD domain is fused in frame with the human IgG1 F105 leader and hinge-CH2-CH3 domains to form CAIX-ECD-Fc. The primers used for cloning CAIX-ECD-Fc are: 5’ tag ggc G GCC CAG CCG GCC cag agg ttg ccc cgg atg ca 3’ (forward) and 5’ tag ggc GCG GCC GC act gct gtc cac tcc agc ag 3’ (reverse). The proteoglycan (PG) domain and carbonic anhydrase (CA) domain were PCR assembled and cloned into pcDNA3.1-hinge-stuffer vector via Nhe I/Not I sites to form CAIX-PG_(1-125)_-Fc or CAIX-CA_(1-41, 131-397)_-Fc fusions. The primers used for cloning CAIX-PG_(1-125)_-Fc are: 5’ tCT AGC TAG CCG CCA CCa tgg ctc ccc tgt gc 3’ (forward) and 5’ tag ggc GC GGC CGC ggg ttc ttg agg atc tcc agg 3’ (reverse). The CA_(1-41, 131-397)_ fragment was generated through two-step PCR. PCR primers for the first step PCR reactions are: To generate CAIX 1-41 with 131-137 overhang, we used 5’ tCT AGC TAG CCG CCA CCA TGg ctc ccc tgt gc 3’ (forward) and 5’GTC ATC CCC TTC TTT GTC CCT ggg caa cct ctg ggg atg ga3’ (reverse); to generate CAIX 131-397, we used 5’ agg gac aaa gaa ggg gat gac 3’(forward) and 5’ tag ggc GCG GCC GC act gct gtc cac tcc agc ag 3’ (reverse). Products from the first PCR reactions were purified and served as the template for the second PCR to make CAIX_(1-41, 131-397)_. The primers are 5’ tCT AGC TAG CCG CCA CCA TGg ctc ccc tgt gc 3’ (forward) and 5’ tag ggc GCG GCC GC act gct gtc cac tcc agc ag 3’ (reverse). The CAIX-ECD domain was also cloned into pcDNA3.1-CD5L-C9 vector at NheI/BspE I sites to form CAIX-ECD_(1-397)_-C9 fusion protein with forward primer 5’ tCT AGC TAG CCG CCA CCA TGg ctc ccc tgt gc 3’ and reverse primer 5’ aag gTC CGG Aac tgc tgt cca ctc cag ca 3’. The soluble CAIX fusion proteins were expressed in 293FT cells through transient transfection with lipofectamine 2000 (Invitrogen), and purified as described above for the scFvFc proteins except the CAIX-ECD-C9 proteins were purified through an 1D4 antibody affinity column and eluted with the C9 peptide.

*- Construction and expression CAIX-PG-GST and CAIX-CA-GST fusion proteins in bacteria*. The CAIX PG_(52-125)_ domain and CA_(121-397)_ domain were amplified by PCR using pcDNA3.1-CD5L-CAIX-C9 as a template and cloned into BamH I and EcoR I sites of the GST fusion expression vector pGEX-3X (1). The primers used for cloning are: PG-forward: 5’-AAA TGA TCA CTg gct ctt ctg ggg aag atg a-3’; PG-reverse: 5’- CGG AAT TCA ggg ttc ttg agg atc tcc ag-3’; CA- forward: 5’- AAA TGA TCA CTg atc ctc aag aac ccc aga a-3’; CA- reverse: 5’-CGG AAT TCA act gct gtc cac tcc agc ag-3’. The product plasmid were transformed into *E. coli* (strain DH5α) and the PG-GST and CA-GST fusion proteins were produced and purified from bacterial culture followed by affinity chromatography purification with Glutathione Sepharose 4B (Amersham Biosciences).

*- Subcloning, expression, and purification of soluble anti-CAIX scFv or scFvFc antibodies*. For soluble scFv production in bacteria, scFv coding sequences were excised from the pFarber phage display vector by NcoI and NotI digestion and inserted into the prokaryotic expression pSyn1 vector (2) in the same orientation. The resulting scFvs with C-terminal c-myc and (His)6 tags were expressed in *E. coli* (TG1 strain) and the soluble scFv antibodies were purified by a nickel-bound chelating Sepharose column (Amersham Bioscience).

For scFvFc expression in mammalian cells, scFv-coding DNA fragments from pFarber phagemid were excised by SfiI/NotI digestion and subcloned into pcDNA3.1-hinge-stuffer, where the anti-CAIX scFv is fused in frame with the human IgG1 hinge-CH2-CH3 domains to form scFvFc fusions. 293FT cells were transfected with anti-CAIX scFvFc expression plasmids by CaPO4 method and allowed to express in 293 SFM II Serum Free Medium (GIBCO) containing 4mM L-Glutamine and 4mM sodium butyrate. The cell culture supernatant containing scFvFc proteins was collected twice every 48 hrs and the scFvFcs were purified by Protein A affinity chromatography.

*Preparation and characterization of CAIX containing paramagnetic proteoliposomes (CAIX-PMPLs) –* Briefly, 1x10^8^ 293T-CAIX cells were lysed with 1% CHAPSO and then incubated with 0.5 x 10^9^ M-280 Dynal beads (Dynal, Lake Success, N.Y.) coated with 1D4 mAb. The protein bound beads were washed intensively and resuspended in buffer containing 1 mg/ml of lipid mixture consisted of POPC/POPE/DOPA (Avanti Polar Lipids, Inc.) at a ratio of 6:3:1 and then dialyzed against buffer containing 100mM (NH_4_)_2_SO_4_, 20mM Tris, pH 7.5, to remove detergent and allow the formation of proteoliposomes.

To evaluate the CAIX-PMPLs protein content, 3x10^7^ proteoliposomes were treated with 2X SDS reducing sample buffer and the eluted sample was separated by SDS-PAGE followed by Coomassie blue staining. The purity of CAIX-PMPLs was also confirmed with PMPLs constructed with [^35^S]-methionine/cysteine (Perkin-Elmer Life Sciences) metabolically labeled 293T-CAIX cell lysates. The radiolabeled “hot” PMPLs were treated as the same method mentioned above and applied for autoradiography analysis.

*Selection of anti-CAIX single-chain antibodies from nonimmune human scFv phage display library with PMPL panning -* The 15 and 12 billion member human scFv-phage display (Mehta I/II) libraries (Mehta and Marasco, unpublished data and see website [www.nfcr-ctae.org](http://www.nfcr-ctae.org/)) were pooled for panning. In brief, 293T cells, CCR5 PMPLs, and CAIX PMPLs were preblocked in blocking buffer (2%BSA/2% non-fat milk/PBS) at 4°C for 30 min. 5x10^12^ plaque-forming unit (pfu) of phage in 3ml of 4% BSA/4% non-fat milk/PBS was first incubated with 2x10^7^ 293T cell and 2x10^7^ CCR5 PMPLs for three separate times to absorb the non-specific clones (4˚C, 30 minutes per absorption). The absorbed phage supernatant was then incubated with 5x10^7^ CAIX PMPLs for 2hrs at 4°C with gentle shaking. Unbound phages were removed by washing with 3ml of PBS containing 0.1% Tween-20. Phage bound to CAIX-PMPLs was eluted by addition of 1ml 100mM triethylamine (TEA) and incubation for 20min at room temperature. The mixture was neutralized with an equal volume of 1 M Tris/HCl, pH 6.8. The PMPLs were pelleted and half of the supernatant was used for phage titration and infection of an exponentially growing culture of *E.coli* TG1 for next round of panning. Two rounds of PMPLs-based panning were performed.

*Cell-based ELISA with phage scFv antibodies -* Single colonies from the first and second rounds of panning were picked randomly and phages were rescued. Cell-based ELISA screening for individual phage-antibody clones was performed with 2x10^5^ 293T-CAIX cells or 293T cells as described (3). Clones were scored positive by ELISA if the O.D.450 for 293T-CAIX cells > five-fold over parental 293T cells.

*Flow cytometric characterization of the anti-CAIX antibody binding activity -* The binding of anti-CAIX antibodies was examined by an indirect immunofluorescence assay. For binding specificity analysis, 5x10^5^ sk-rc-52 (CAIX positive) and sk-rc-59 (CAIX negative) cells as well as 293T-CAIX/293T cells (data not shown) were incubated with 1µg/50ul each scFvFc antibody antibody for 45 mins at 4°C followed by staining with FITC- or PE-conjugated goat anti-human IgG. For titration analysis, 5x10^5^ 293T-CAIX cells were incubated with each serially diluted scFvFc antibodies followed by secondary staining. Cells were washed in PBS containing 0.5% BSA and analyzed on FACScan apparatus. The EC50 [concentration of antibody that reaches half-maximal for percent cell binding or geometric mean fluorescence intensity (GMFI), respectively] were used as a measure of the relative binding affinity of each scFvFc to CAIX positive cells.

*Kinetic analysis of binding interactions between anti-CAIX scFvFcs and CAIX-ECD-C9 by Surface Plasmon Resonance (SPR) -* The kinetic interactions between anti-CAIX scFv-Fcs and CAIX-ECD-C9 were measured by SPR using Biacore T100. Sensor chip CM4, amine coupling reagents [N-ethyl-N’-dimethylaminopropyl carbodimide, EDC; N-hydroxysuccinimide, NHS; and 1M ethanolamine HCl (pH 8.5)], human antibody capture kit and HBS-EP+ buffer were obtained from GE Healthcare.  *- Sensor surface preparation*. Kinetic interaction studies were performed via a capture technique. Monoclonal mouse anti-human IgG (Fc), the capture antibody, was amine coupled on a CM4 chip as per the instructions provided with the human antibody capture kit. scFv-Fc antibodies served as the ligands. Three different scFv-Fc antibodies were captured on the chip in three flow cells - Fc2, Fc3 and Fc4 - sequentially, per analyte binding cycle in each assay. Flow cell (Fc) 1 that was prepared similarly as the other surfaces served as the reference and blank buffer control surface for the binding assays and lacked a captured ligand. The analyte, namely the purified CAIX-ECD-C9 antigen, was passed serially through all the flow cells, including the control flow cell, Fc1.

- *Interaction analysis*. The ligand and analyte were diluted in the running buffer HBS-EP+. Typically, the ligand density captured for each analyte binding cycle was equivalent to Rmax 50. The CAIX-ECD-C9 analyte was injected over all the four flow cells, in a series of 7 different concentration cycles with 2-fold dilution of the maximum concentration which varied from 50nM-400nM, depending on the affinity of the antibodies being investigated. One concentration was duplicated. There were a total of 13 binding cycles including 3 start-up cycles followed by ten analyte-binding cycles consisting of 8 cycles of all concentration tested and two 0 nM analyte injections at either end. The fitted data sets shown in the sensograms have 5 different concentrations of the analyte diluted serially from 50, 100 or 400nM for the different antibodies based on their affinity (50nM- G119, G10, G37; 100nM- G106, G36, G39, G57, G45, G40, G27; 400nM- G6, G125, G9). The analyte was injected at a flow rate of 45ul/min and the association and dissociation phases were monitored for 2 and 5min, respectively. The anti-human Fc surface was regenerated after each binding cycle by a 45s pulse of 3M MgCl_2_ provided with the human antibody capture kit. The screening of all the antibodies was performed at an analysis temperature of 25^o^c. Kinetic assays for each antibody were performed in three different experiments. Non-specific binding of the analyte to the reference anti-human Fc surface was absent.

**References used in** **Supplemental Material and Methods**

1. Smith, D. B., and Johnson, K. S. (1988) *Gene* **67**(1), 31-40

2. Schier, R., Marks, J. D., Wolf, E. J., Apell, G., Wong, C., McCartney, J. E., Bookman, M. A., Huston, J. S., Houston, L. L., Weiner, L. M., and et al. (1995) *Immunotechnology* **1**(1), 73-81

3. Xu, C., Sui, J., Tao, H., Zhu, Q., and Marasco, W. A. (2007) *J Immunol* **179**(4), 2408-2418
